# Chemogenetic manipulation of astrocyte activity at the synapse— a gateway to manage brain disease

**DOI:** 10.3389/fcell.2023.1193130

**Published:** 2023-07-18

**Authors:** Maria João Pereira, Rajagopal Ayana, Matthew G. Holt, Lutgarde Arckens

**Affiliations:** ^1^ Department of Biology, Laboratory of Neuroplasticity and Neuroproteomics, KU Leuven, Leuven, Belgium; ^2^ KU Leuven Brain Institute, Leuven, Belgium; ^3^ Instituto de Investigação e Inovação em Saúde (i3S), Laboratory of Synapse Biology, Universidade do Porto, Porto, Portugal

**Keywords:** astrocytes, synaptogenesis, synaptic plasticity, heterogeneity, DREADDs, CNS disease

## Abstract

Astrocytes are the major glial cell type in the central nervous system (CNS). Initially regarded as supportive cells, it is now recognized that this highly heterogeneous cell population is an indispensable modulator of brain development and function. Astrocytes secrete neuroactive molecules that regulate synapse formation and maturation. They also express hundreds of G protein-coupled receptors (GPCRs) that, once activated by neurotransmitters, trigger intracellular signalling pathways that can trigger the release of gliotransmitters which, in turn, modulate synaptic transmission and neuroplasticity. Considering this, it is not surprising that astrocytic dysfunction, leading to synaptic impairment, is consistently described as a factor in brain diseases, whether they emerge early or late in life due to genetic or environmental factors. Here, we provide an overview of the literature showing that activation of genetically engineered GPCRs, known as Designer Receptors Exclusively Activated by Designer Drugs (DREADDs), to specifically modulate astrocyte activity partially mimics endogenous signalling pathways in astrocytes and improves neuronal function and behavior in normal animals and disease models. Therefore, we propose that expressing these genetically engineered GPCRs in astrocytes could be a promising strategy to explore (new) signalling pathways which can be used to manage brain disorders. The precise molecular, functional and behavioral effects of this type of manipulation, however, differ depending on the DREADD receptor used, targeted brain region and timing of the intervention, between healthy and disease conditions. This is likely a reflection of regional and disease/disease progression-associated astrocyte heterogeneity. Therefore, a thorough investigation of the effects of such astrocyte manipulation(s) must be conducted considering the specific cellular and molecular environment characteristic of each disease and disease stage before this has therapeutic applicability.

## Introduction

The central nervous system (CNS) is a highly diverse cellular environment, where neurons are surrounded by a multitude of cell types, including astrocytes, microglia, oligodendrocytes, and ependymal cells, which are collectively known as glial cells. For many years, brain function and behavioral output were thought to depend exclusively on neuronal circuit activity, while glial cells were merely regarded as supportive cells. However, it is now recognized that glial cells actively communicate with neurons and modulate neuronal activity.

Among glial cells, astrocytes are the most abundant and are known to be indispensable for correct brain function, as they actively participate in synapse formation, plasticity and function, as well as controlling blood-brain barrier permeability and blood flow, providing metabolic support to neurons and modulating neuroinflammation (Sofroniew and Vinters, 2009). Indeed, astrocytes possess a highly branched, “star-shaped” morphology, enabling them to contact thousands to millions of synapses via peripheral astrocytic processes (Semyanov and Verkhratsky, 2021; Holt, 2023; Salmon et al., 2023). The discovery that astrocytes and neurons communicate with one another at the synapse to modulate synaptic transmission led to the concept of the tripartite synapse (Parpura et al., 1994; Pasti et al., 1997; Bezzi et al., 1998; Newman and Zahs, 1998; Araque et al., 1999).

Upon release from the neuronal pre-synaptic element, neurotransmitters activate neurotransmitter receptors expressed by astrocytes, including G protein-coupled receptors (GPCRs). A rise in astrocytic intracellular Ca^2+^ typically follows, which can then lead to the subsequent (local) release of gliotransmitters and other neuroactive molecules capable of modulating synaptic activity and plasticity (Perea et al., 2009). Importantly, astrocytes can also communicate with each other via gap junctions. This enables locally induced Ca^2+^ signals to propagate to neighbouring astrocytes, allowing these cells to also coordinate the activity of otherwise distant synapses (Giaume et al., 2021).

Given the wide array and range of astrocytic functions, particularly at the tripartite synapse, it is not surprising that astrocytic dysfunction has long been implicated in the pathogenesis of several CNS diseases, including neurodevelopmental, neuropsychiatric and neurodegenerative diseases, making these cells attractive therapeutic targets. In this review, we will first provide a general overview of the involvement of astrocytes in synapse formation, activity and plasticity in development and adulthood. Next, we will shed light on how chemogenetics, a technique using genetically engineered GPCRs to modulate astrocytic activity, induces astrocytic Ca^2+^ signalling and gliotransmitter release, mimicking, to a certain degree, endogenous signalling pathways. Finally, we will finish by discussing how this technique might reveal new molecular pathways that can be exploited therapeutically in the future.

## Astrocytes control neuronal circuit development

The generation of fully functional neuronal circuits capable of receiving, integrating, and responding to a wide variety of intrinsic and extrinsic stimuli largely depends on the establishment of proper synaptic connections. Synaptogenesis begins when the terminal bouton of a neuron comes into close contact with tiny protrusions, known as spines, on the dendrites of another neuron. Both the shape and size of spines are vital to ensuring adequate synaptic transmission. Thinner spines are considered more unstable and are associated with a more immature or silent state, as they often lack the post-synaptic machinery necessary for synaptic transmission. Once physical contact has been established, spines can undergo maturation. During this process, the spine head enlarges, spines acquire a post-synaptic density (PSD) and neurotransmitter receptors accumulate in the post-synaptic membrane, forming larger mushroom-like spines, and leading to increased synaptic potency (Yoshihara et al., 2009; Xu et al., 2020). During post-natal brain development, critical periods are pivotal to uniquely shaping the CNS. During these periods of heightened experience-dependent circuit remodeling, stable synapses are formed at a high rate. Experience acts by strengthening the relevant ones, which are eventually integrated into the circuit. On the other hand, redundant spines weaken over time, due to lack of relevant stimulation, and are eventually eliminated. This maturation process changes both the cell-cell connections and functional output of an excitatory network producing a more stable and mature circuit. Astrocytes have been identified as key regulators of critical period closure, ensuring proper brain wiring (Ackerman et al., 2021; Ribot et al., 2021). In adulthood, synapses are much more stable but synaptic remodelling and plasticity still occur to enable circuit adaptations to new experiences, driving learning and memory formation, and recovery from CNS injury and disease (Pascual-Leone et al., 2005; Hübener and Bonhoeffer, 2014). Therefore, synaptic assembly and circuit refinement must be tightly regulated as aberrant synapse formation is thought to contribute, for example, to the emergence of neurodevelopmental diseases (Washbourne, 2015).

**FIGURE 1:**
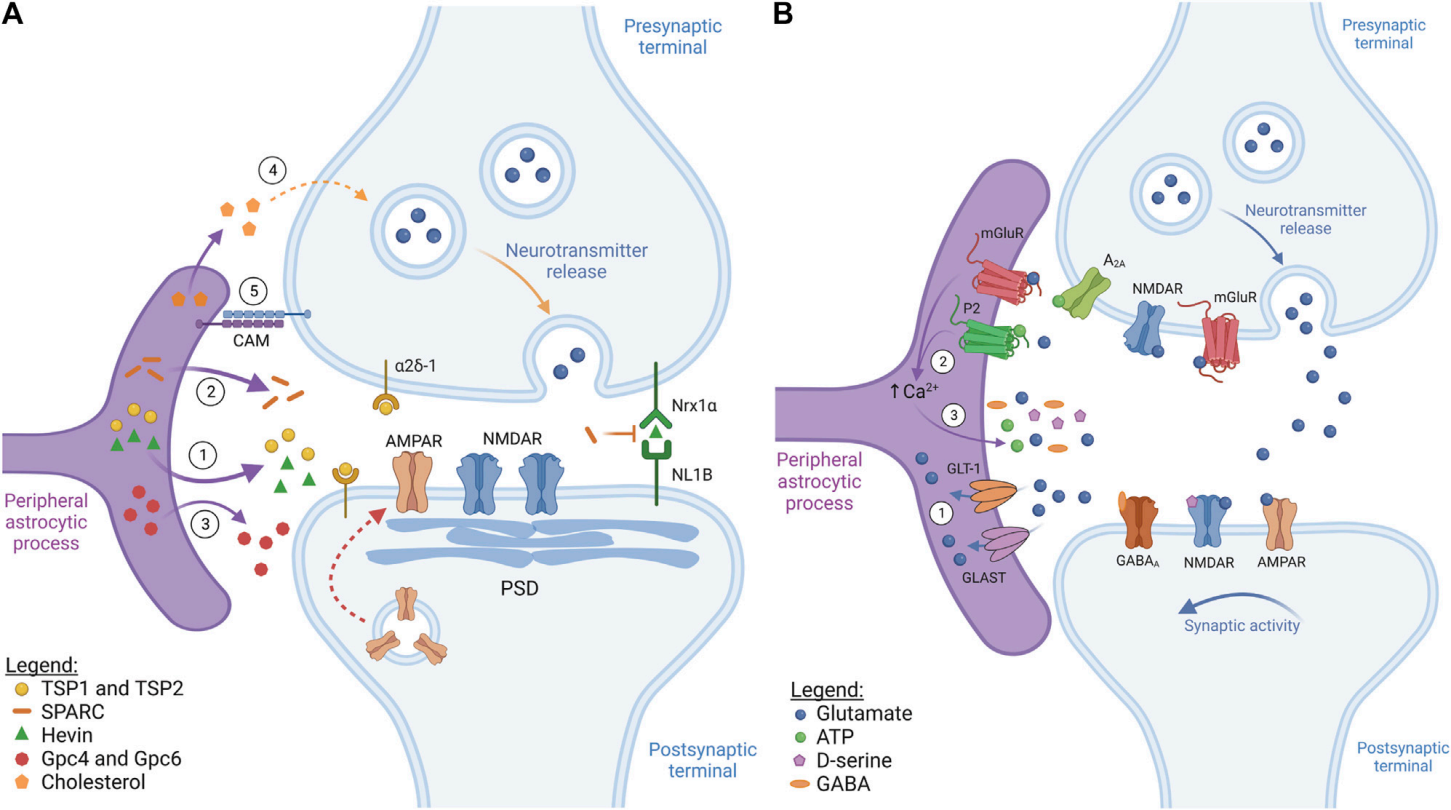
Astrocytes control synapse formation and plasticity (figure represents a general synapse). **(A)** Synaptogenesis. ① During early development, astrocytes secrete pro-synaptogenic factors thrombospondin 1 and 2 (TSP1 and TSP2) and hevin. TSP1 and TSP2 interact with the neuronal receptor α2δ-1, while hevin bridges neuronal neurexin-1α (NRX1α) and neuroligin-1B (NL1B), inducing structural synapse formation. These factors induce the formation of immature synapses containing synaptic vesicles, post-synaptic density (PSD) and NMDARs, but lacking AMPARs. ② Astrocytes can also secrete a hevin antagonist, SPARC, which inhibits hevin-induced synaptogenesis, controlling the rate of new synapse formation. ③ Astrocyte-secreted molecules, such as glypican 4 and 6 (Gpc4 and Gpc6), contribute to synapse maturation by recruiting AMPARs to the post-synaptic membrane (red dotted arrow). ④ Astrocyte-secreted cholesterol is also crucial during synaptic maturation as it regulates pre-synaptic vesicle exocytosis. ⑤ Astrocyte-neuron cell adhesion molecules (CAM), like protocadherins, provide stability and promote synaptic development via contact-mediated signalling. **(B)** Synaptic transmission and plasticity. ① Once released, neurotransmitters stimulate mainly ionotropic receptors at the post-synaptic neuron to propagate/suppress synaptic transmission. Following this, specialized transporters, like GLT-1/GLAST, take up excess neurotransmitter, such as glutamate, thus preventing excitotoxicity. ② Neurotransmitters released at the synapse also bind and activate astrocytic metabotropic neurotransmitter receptors, such as mGluR and purinergic P2 receptors, which commonly induces astrocytic Ca^2+^ levels to rise. ③ Synaptically-evoked Ca^2+^ increases usually contribute, at least in part, to gliotransmitter release (glutamate, ATP, D-serine, GABA). These gliotransmitters interact with neuronal receptors at the pre- and post-synaptic elements, regulating synaptic activity and affecting neurotransmitter release.

Synaptogenesis greatly increases following astrocyte differentiation (Freeman, 2010) and the expansion in astrocyte numbers that occurs during the first post-natal week (Ge et al., 2012). Furthermore, synaptogenesis also appears concomitant to astrocyte structural maturation (Clavreul et al., 2019). Both astrocyte-secreted molecules and astrocyte-expressed cell adhesion proteins appear to be important factors in the process (Figure 1A). While progress has been made based on studies focusing on factors mediating excitatory synaptogenesis, it is likely that many other factors are still to be identified. In contrast, inhibitory synapse formation is not yet well understood (Um, 2017). Synapse formation can generally be regarded as a two-stage process. First, structural synapse formation takes place which is then followed by functional synapse maturation.

To induce the structural assembly of glutamatergic synapses, astrocytes secrete the pro-synaptogenic factors thrombospondin 1 and 2 (TSP1 and TSP2) (Christopherson et al., 2005) and hevin (Figure 1A) (Kucukdereli et al., 2011; Risher et al., 2014; Singh et al., 2016). TSP1 and TSP2 act by binding the neuronal receptor α2δ-1 (Eroglu et al., 2009), while hevin promotes synapse assembly by bridging neuronal neurexin-1α (Nrx1α) and neuroligin-1B (NL1B) (Singh et al., 2016). These synapses usually possess synaptic vesicles, active release sites and PSD. However, they are functionally inactive because, even though they possess post-synaptic N-methyl-D-aspartate glutamate receptors (NMDAR), they lack α-amino-3-hydroxy-5-methyl-4-isoxazolepropionic acid glutamate receptors (AMPAR) and the release of synaptic vesicles is suboptimal (Christopherson et al., 2005).

To produce functionally mature synapses, astrocytes then secrete factors, such as the heparan sulfate proteoglycans glypican 4 and glypican 6 (Gpc4 and Gpc6), which increase the expression of the GluA1 subunit of AMPAR at the post-synaptic terminal (Figure 1A) (Allen et al., 2012). Additionally, astrocyte-secreted Chordin-like 1 (Chrdl1) has been shown to increase the levels of GluA2-containing AMPAR to the synapse, leading to Ca^2+^ impermeability of this ionotropic receptor and contributing to the maturation of excitatory glutamatergic synapses (Brill and Huguenard, 2008; Blanco-Suarez et al., 2018). Astrocytic tumor necrosis factor-alpha (TNF-α) can also recruit AMPAR to excitatory synapses, while decreasing GABA_A_ receptor density at inhibitory synapses, thus regulating overall neuronal circuit activity (Beattie et al., 2002; Stellwagen et al., 2005; Stellwagen and Malenka, 2006). Astrocyte-derived cholesterol is also crucial for proper synaptic maturation by regulating pre-synaptic vesicle exocytosis (Figure 1A). These effects have been described both *in vitro* and *in vivo* (Mauch et al., 2001; Pfrieger, 2003; van Deijk et al., 2017). For instance, in the hippocampus of mice with reduced cholesterol production, the total vesicle pool and the number of synaptic vesicles ready for release at the pre-synapse are reduced, which is accompanied by a reduction in the levels of SNAP-25, a protein necessary for vesicle fusion (van Deijk et al., 2017).

In addition to the factors which have a positive impact on synaptogenesis, astrocytes can also release secreted protein acidic and rich in cysteine (SPARC), which is an antagonist of hevin (Figure 1A). Thus, SPARC negatively affects synapse development by counteracting hevin-mediated synaptogenesis, likely by competitively interacting with the same neuronal proteins as hevin (Kucukdereli et al., 2011). Furthermore, SPARC has been shown to prevent the overaccumulation of AMPAR receptors at the excitatory post-synaptic membrane by destabilizing β3-integrin complexes (Jones et al., 2011), which are important regulators of AMPAR stability at the synapse (Cingolani et al., 2008).

Synapse development and stability are further controlled by astrocyte-neuron adhesion proteins (Figure 1A), which have been extensively reviewed (Tan and Eroglu, 2021). For instance, cultured embryonic retinal ganglion cells seem to require direct contact with astrocytes to form mature synapses, suggesting coordinated actions between secreted and contact-mediated signals in driving synaptogenesis (Barker et al., 2008). One of the most common examples of contact-mediated synaptogenesis is the one mediated by γ-protocadherins. This family of cell adhesion proteins is expressed at the tripartite synapse by neurons and astrocytes alike (Phillips et al., 2003; Garrett and Weiner, 2009) and has been shown to be essential for excitatory and inhibitory synapse formation *in vitro* and *in vivo* (Garrett and Weiner, 2009). Astrocyte development and, consequently, synaptogenesis are also controlled by the interaction of astrocytic neuroligins (NL) with neuronal neurexins (Nrx): knockdown of astrocytic NL2 decreases excitatory synapse formation and function while promoting inhibitory synaptic function (Stogsdill et al., 2017). These dynamics are likely very complex as astrocyte-neuron co-cultures only fully mature when both cell types are from the same brain area, implying a degree of regional specialization in astrocyte-neuronal interactions (Morel et al., 2017).

Synaptic pruning is also an essential part of neuronal circuit formation. Astrocytes can balance out their synaptogenic properties by modulating synapse elimination, thus preventing excessive accumulation of superfluous synapses. Astrocytes are phagocytic cells and express the receptors MEGF10 and MERTK. These receptors recognize phosphatidylserines at the surface of target synapses as opsonic signals, leading to their degradation (Chung et al., 2013; Scott-Hewitt et al., 2020). Furthermore, astrocyte-microglia crosstalk also contributes to synaptic pruning. For example, astrocytic IL-33 has been shown to induce microglia-mediated synaptic pruning, although the downstream mechanisms that trigger the microglial response are still unclear (Vainchtein et al., 2018).

It is likely that not all astrocytes have the same secretory phenotype, as they have been described as a rather heterogeneous cell population (Ben Haim and Rowitch, 2017; Khakh and Deneen, 2019; Batiuk et al., 2020). Astrocytic factors have specialized functions to ensure correct circuit maturation and different factors seem to be necessary for the formation and maturation of specific subtypes of synapses. Therefore, astrocytes in different (sub)regions of the brain could be specialized in secreting specific factors which are crucial for synapse formation in that region. For example, astrocyte-secreted hevin appears to be crucial for thalamocortical excitatory synapse formation (Risher et al., 2014). Chrdl1 expression is enriched particularly in upper cortical layers and in striatal astrocytes, indicating that its actions may be restricted to these brain areas (Blanco-Suarez et al., 2018). Astrocytes originating from the dorsal or ventral spinal cord have different gene expression profiles and both seem to be essential to synaptogenesis (Tsai et al., 2012; Molofsky et al., 2014). For example, the elimination of ventral astrocytes expressing *Sema3a* compromises motor and sensory neuron circuit formation (Molofsky et al., 2014). Hence, synaptogenesis appears to require a complex interplay of astrocytic molecules and signals that must be tightly coordinated to control circuit formation and refinement *in vivo*, which is far from being completely understood (Holt, 2023). Phagocytic capacity may also vary between astrocyte populations and brain regions. More detailed knowledge about these aspects of astrocyte physiology and function will be necessary to be able to phenocopy or boost astrocyte function as a therapy for brain disease in the future.

## Astrocyte-neuron interactions regulate synaptic transmission and plasticity

Functional, mature synapses continuously transfer information between neurons via the release of neurotransmitters, neuropeptides and neuromodulators. The remarkable discovery that astrocytes are also active participants in synaptic transmission, responding to and controlling neuronal excitability and synaptic plasticity through various mechanisms established the concept of the tripartite synapse (Araque et al., 1999) (Figure 1B).

Astrocytes express a wide variety of neurotransmitter transporters, including glutamate transporters, such as the glutamate transporter 1 (GLT-1) and glutamate/aspartate transporter (GLAST) (Figure 1B), and GABA transporters, such as GAT1 and GAT3, which take up their respective neurotransmitters from the synaptic cleft and extrasynaptic space following synaptic transmission. This restricts neurotransmitter action, preventing excitotoxicity which would lead to synaptic dysfunction (Ishibashi et al., 2019; Mahmoud et al., 2019).

Astrocytes also express a wide array of neurotransmitter receptors (Figure 1B). Neurotransmitters thus not only act by stimulating or inhibiting the pre- and post-synaptic neuronal elements but also control astrocytic activity. Most astrocytic neurotransmitter receptors consist of GPCRs, including metabotropic glutamate receptors (mGluR), purinergic receptors (P2Y), and the GABA_B_ receptor (Liu et al., 2021). Activation of these receptors typically triggers Ca^2+^ signals in astrocytes via the phospholipase C (PLC)-IP_3_ pathway. Briefly, upon activation by their ligand, many GPCRs, such as Gq-coupled mGluRs, stimulate PLC to form IP_3_ which then induces Ca^2+^ release from the endoplasmic reticulum via activation of IP_3_ receptors (IP_3_R) (Volterra et al., 2014; Shigetomi et al., 2016). Depending on their nature and intensity, Ca^2+^ signals can then propagate to neighbouring cells via gap junctions, thus highlighting the complexity of Ca^2+^ signals in astrocytes.

Astrocytes seem to discriminate activity originating not only from different brain regions but also from different neuronal subtypes within the same region. As revealed by astrocytic Ca^2+^ imaging, hippocampal astrocytes from the *stratum oriens* of the CA1 respond to cholinergic but not glutamatergic inputs originating in the alveus (Araque et al., 2002), whereas the same astrocytes respond to glutamate if the signal originates from the Schaffer collateral (Perea and Araque, 2005). In the barrel cortex, astrocytes in layer 2/3 increase intracellular Ca^2+^ levels in response to glutamatergic stimuli from layer 4, but not from layer 2/3 (Schipke et al., 2008). A single astrocyte simultaneously contacts thousands of synapses, which may be excitatory or inhibitory, making it highly likely that astrocyte processes associated with different types of synapses express different receptors (Holt, 2023). Hence, astrocytes can respond to neuronal activity in a highly intricate way that is cell-, region-, and pathway-specific.

Astrocytes are thought to respond to neuronal activity by releasing small neuroactive molecules (gliotransmitters), including glutamate, GABA, D-serine, and ATP, which can in turn modulate synaptic activity (Figure 1B). A variety of mechanisms for gliotransmitter release have been proposed. Vesicular gliotransmitter release has been widely proposed and is thought to be controlled, at least in part, by synaptically-evoked increases in intracellular astrocyte Ca^2+^ (Perea et al., 2009). However, alternative mechanisms have also been proposed, not all of which are Ca^2+^-dependent, such as diffusion through conductance pores opened following P2X_7_ activation by ATP and swelling-activated anion channels, or even diffusion through gap junction hemichannels (Hamilton and Attwell, 2010). Once released, gliotransmitters can contribute to regulate neuronal excitability and synaptic plasticity by inducing long-term potentiation (LTP) or long-term depression (LTD). LTP results in synaptic strengthening by increasing synaptic responses, while LTD weakens synapses due to decreasing synaptic responses (Turrigiano, 2008). In hippocampal dentate granule cells, astrocyte-released glutamate activates NMDARs in the afferent neurons, prolonging excitatory synaptic transmission (Jourdain et al., 2007), and induces AMPAR-mediated spontaneous excitatory synaptic current in CA1 pyramidal neurons (Fiacco and McCarthy, 2004). Additionally, astrocytic glutamate can activate pre-synaptic mGluRs, which enhances NMDAR-mediated currents in CA1 hippocampal neurons, but inhibits CA3 hippocampal neurons (Grishin et al., 2004). In addition to glutamate, NMDAR activation also requires D-serine binding. Astrocyte-released D-serine in CA1 induces NMDAR-dependent LTP, controlling the synaptic plasticity of neighboring excitatory synapses (Henneberger et al., 2010). Furthermore, ATP and its degradation product adenosine interact with A_1_ and A_2A_ pre-synaptic receptors, either enhancing or inhibiting neuronal excitability. In hippocampal CA1, astrocyte activation via mGluR5 leads to ATP release, activating A_2A_ receptors and increasing synaptic transmission and LTP (Panatier et al., 2011). On the other hand, in hippocampal slices, GABA_B_ receptor-mediated astrocyte activation induces both glutamate and ATP release. An initial excitatory response is driven by glutamate, followed by the ATP response which induces synaptic depression (Covelo and Araque, 2018). These studies provide evidence that gliotransmitters may facilitate or inhibit neuronal excitability depending on the brain (sub)region and type of receptors expressed and activated. This might be further influenced by the temporally segregated co-release of different gliotransmitters by the same, or even different astrocytes, thus emphasizing the intricate nature of astrocyte-neuron communication.

Proper synaptic function and plasticity are vital to drive animal behavior and support complex brain processes. The role of astrocytes in, for example, memory consolidation has been highlighted by observations that in IP_3_R2 knock-out mice, in which Ca^2+^ signalling in astrocytes is impaired, synaptic plasticity is compromised (Takata et al., 2011; Chen et al., 2012; Navarrete et al., 2012). Furthermore, Halassa and colleagues showed that blockage of astrocyte vesicular release affects memory formation (Halassa et al., 2009), while Stehberg and colleagues revealed that administration of a mixture of gliotransmitters rescued memory loss (Stehberg et al., 2012). Even though the exact roles astrocytes play in such processes still remain elusive, these studies point towards a path in which specifically manipulating astrocytic activity holds promise to allow in-depth characterization of astrocyte roles, not only in learning and memory but also in other high-order brain functions and behaviors.

Finally, just like during brain development, synapses in the adult brain are also subject to ongoing pruning by astrocytes, using similar molecular machinery to help maintain circuit homeostasis and facilitate processes like learning and memory formation (Lee et al., 2021).

## Chemogenetic manipulation of astrocyte activity

Given the significance of astrocytes in synapse formation and function (including plasticity), there has been a growing interest to manipulate their activity to modulate neuronal activity and positively impact CNS health. Chemogenetics is a widely used technique, based on genetically engineered proteins, which have been specifically modified to respond to otherwise inert synthetic molecules, instead of their endogenous ligands. Since GPCRs comprise the main group of receptors activating astrocytes, chemogenetics commonly uses engineered GPCRs known as Designer Receptors Exclusively Activated by Designer Drugs (DREADDs) (Armbruster et al., 2007).

Target genes, such as DREADDs, can be expressed as transgenes in genetically manipulated mouse lines, by using vectors [such as those based on adeno-associated virus (AAV) or lentivirus (LV)] injected into target brain regions or intravenously (e.g., retro-orbital and tail injections), or even by resorting to *in utero* (IUE) or post-natal (PALE) electroporation. By combining any of these approaches with astrocyte-specific promoters, expression of the gene of interest can be restricted to this cell population. When choosing between one of these methods to study astrocyte function, one should not only take the goal of the experiment into account but also the advantages and disadvantages inherent to each approach (Table 1). Many transgenic mouse lines, mostly tamoxifen-inducible Cre lines (Cre/ERT2), are particularly suitable to study the impact of astrocytes on brain-wide function. However, high rates of efficiency and specificity are sometimes difficult to accomplish since astrocyte gene expression highly depends on several factors such as developmental stage and brain region, and genes regarded as astrocytic markers may also be expressed in other cell types, including neural progenitor cells (Yu et al., 2020a). The *Aldh1l1*-Cre/ERT2 and the *Fgfr3*-Cre/ERT2 mouse lines are, to date, some of the mouse lines which have achieved the highest rates of efficiency and specificity (Young et al., 2010; Srinivasan et al., 2016; Winchenbach et al., 2016; Yu et al., 2021), while others, such as the *Slc1a3*-Cre/ERT2 (Slezak et al., 2007; Srinivasan et al., 2016), *S100β*-Cre (Tanaka et al., 2008), and *GFAP*-Cre/ERT2 (Casper et al., 2007; Park et al., 2018), have been described to target fewer astrocytes and to have more off-target effects than the *Aldh1l1*-Cre/ERT2 mouse line. Importantly, by crossing *GFAP*-Cre/ERT2 mice with Cre-responsive Rosa-CAG-lox-hM3Dq, an inducible DREADD mouse line was successfully created in which the DREADD construct (hM3Dq) was specifically expressed in the soma and processes of *Gfap*-positive glial cells (Sciolino et al., 2016). Intravenous injection of AAV-PHP.eB, containing the *GfaABC*_*1*_ *D* promoter, consistently and specifically targets high amounts of astrocytes across the entire brain, thus providing a valuable alternative to the use of transgenic mouse lines (Chan et al., 2017; Challis et al., 2019). While is it true that brain-wide specific astrocyte targeting can provide valuable information about the contribution of these cells to global brain function, the emerging evidence that astrocytes show inter- and intra-regional heterogeneity, and appear to be matched to local circuits responsible for generating particular behaviors, makes it increasingly important to tackle the role of astrocyte (subpopulations) in distinct brain areas (Nagai et al., 2021). Given the limited diffusion capacity of certain AAV and LV vectors (Scheyltjens et al., 2015), region-specific astrocyte targeting is mostly accomplished by performing intracranial viral vector injections. Namely, AAV2/5 and AAV2/9, containing the *GfaABC*_1_ *D* promoter, are commonly used as this has been described to achieve relatively high rates of efficiency (Nagai et al., 2019: 84%; Yu et al., 2018: 89%) and specificity (Vagner et al., 2016; Octeau et al., 2018; Yu et al., 2018; Nagai et al., 2019; Yu et al., 2021). Similarly, LV, such as the Mokola pseudotyped LV (Droguerre et al., 2019), can also be used to locally target astrocytes with the advantage that they can transport more genetic material than AAVs (Cannon et al., 2011; Droguerre et al., 2019). However, LVs have been associated with the risk of mutagenesis since they integrate into the genome. IUE provides yet another alternative to express genes of interest in brain cells and has been successfully implemented in several animal models such as mice, rats, ferrets and cats (Yamashiro et al., 2022; Edwards-Faret et al., 2023). As astrocytes start developing in the CNS shortly after birth, PALE, an adaptation of IUE, can be used to specifically target astrocytes (García-Marqués and López-Mascaraque, 2013; Gee et al., 2015; Stogsdill et al., 2017; Kittock and Pilaz, 2023). In this technique, plasmids, upon injection into the parenchyma of newborn mouse pups (P0-P1), are delivered into the cells due to the action of electrical impulses which disrupt the cell membrane, allowing the passage of the DNA. Besides being less damaging, since the manipulation is performed early in life, and allowing the transfection of bigger constructs than the viral vector approaches, by precisely controlling the injection site and/or the position of the electrodes, both IUE and PALE allow region specific transgene expression (Yamashiro et al., 2022; Kittock and Pilaz, 2023). IUE and PALE have been used to express DREADDs in neurons (Hurni et al., 2017; Muthusamy et al., 2017). Although this has yet to be accomplished for astrocytes, the positive results obtained for neurons hold great promise to also take advantage of this technique to target DREADDs to astrocytes when using the appropriate promoters. It is interesting to note that the above-mentioned approaches can also be combined for better results regarding specificity and expression levels. For instance, astrocyte-specific Cre lines, such as *Aldh1l1*-CreERT2, generally make use of full-length promoters and are, therefore, generally considered to more faithfully recapitulate the endogenous gene expression profile. Combining the use of such Cre-lines with injections of viral vectors (or plasmids) that express DREADDs in a Cre-inducible manner, allows transgene expression under the control of strong ubiquitous promoters, such as the cytomegalovirus (CMV) promoter, which often results in higher expression levels (although potentially enhanced toxicity cannot be discounted). Additionally, combining IUE/PALE with the use of DREADDs could hold the potential to unravel the roles of astrocytes in non-model organisms, as well as species with a more complex brain structure than mice. However, none of the approaches mentioned above take the matter of intra-regional diversity into account for which vectors and/or transgenic lines containing promoters for specific astrocyte subpopulations should be used. Even though the existence of such vectors and transgenic lines is not yet a reality, the recent generation of single-cell/nucleus RNA sequencing data sets might help propel the development of such tools and even the use of intersectional genetics (Beckervordersandforth et al., 2010). This will be crucial to determine the role of specific subpopulations in defined brain regions (Huang et al., 2020). As described below, to date, DREADDs have been successfully expressed in astrocytes, under the control of the *GFAP* promoter, in both transgenic mouse models and AAV systems, and it is likely that such techniques can be modified to use promoters targeting astrocyte subpopulations (Bang et al., 2016; Shen et al., 2021).

**TABLE 1:** Possible strategies for brain-wide and region-specific DREADD targeting. *Indicates specific approaches which have been successfully implemented to express DREADDs specifically in astrocytes. †Indicates strategies which have been successful in expressing DREADDs specifically in neurons but have yet to be tested for astrocytes. The remaining approaches have been previously used to (non-specifically) target astrocytes and not in the specific context of DREADD expression.

Technical approach	Astrocyte targeting	Expression system	Advantages	Limitations
Transgenic mouse lines	Brain-wide	Constitutive expression: *GFAP-hM3Dq (Agulhon et al., 2013), *S100* β -Cre (Tanaka et al., 2008), *GFAP*-Cre (Zhuo et al., 2001)	No capacity limitation (bigger promoters may be used); Little to no invasiveness.	Promoter-dependent efficiency and/or specificity; Possibility of off-target, systemic effects; Inducible lines require tamoxifen administration; Time-consuming and expensive.
		Inducible expression: *Aldh1l1*-Cre/ERT2 (Srinivasan et al., 2016; Winchenbach et al., 2016; Yu et al., 2021), *Fgfr3-*Cre/ERT2 (Young et al., 2010), *Slc1a3*-Cre/ERT2 (Slezak et al., 2007; Srinivasan et al., 2016), *GFAP-Cre/ERT2 (Casper et al., 2007; Park et al., 2018) crossed with Rosa-CAG-lox-hM3Dq (Sciolino et al., 2016)		
Intravenous AAV injections (blood-brain barrier crossing)	Brain-wide	AAV-PHP.eB; *GfaABC1D* promoter (Challis et al., 2019; Chan et al., 2017), scAAV9; chicken β-actin hybrid promoter (CB) promoter (Foust et al., 2009), and *GfaABC*_1_ *D* promoter (Dashkoff et al., 2016), AAV-rh10; cytomegalovirus (CMV) promoter (Tanguy et al., 2015)	High efficiency and specificity; Low invasiveness.	Limited packaging capacity (AAV ≤5 kb; small promoters); Requires high vector load (particularly AAV9 and AAV-rh10) which may trigger immune responses; Possibility of off-target, systemic effects; Expensive.
Intracranial AAV or LV microinjections	Region-specific	*AAV2/5 and *AAV2/9; *GfaABC1D* promoter (Hennes et al., 2020; Nagai et al., 2019; Octeau et al., 2018; Vagner et al., 2016; Yu et al., 2018, 2021), Mokola pseudotyped LV (Droguerre et al., 2019)	Relatively high efficiency and specificity; Limited diffusion capacity is advantageous to restrict vector expression to astrocytes in the target region.	Limited packaging capacity (AAV ≤5 kb and LV ≤10 kb; small promoters); Invasive; Limited diffusion capacity is disadvantageous if the region of interest is big or if brain-wide labelling is desired; Expensive.
*In utero* (IUE) and post-natal (PALE) electroporation	Region-specific	IUE: †Ubi-hM3Dq-GFP (Hurni et al., 2017), mGFAP kmyrTdTomato; misPiggy plasmid system (Edwards-Faret et al., 2023)	Less damaging since the young brain is more plastic and recovers better from insults than the adult brain; No limitation in transgene size; Robust expression; Applicable to many species, including non-model organisms.	Unsuitable to study proliferating cells unless in combination with techniques such as the *piggyBac* transposon system; Limited coverage; Difficult to control the targeted area; Possibility of cell death due to high-voltage electrical pulses.
		PALE: †pAAV-*pSYN-DIO-HA- hM4D(Gi)-IRES-mCitrine* (Muthusamy et al., 2017)*,* pZac2.1-gfaABC1D-EGFP, pZac2.1-gfaABC1D-mCherry-CAAX (Stogsdill et al., 2017)		

The modified human M3 and M4 muscarinic receptors coupled to Gq or Gi proteins (hM3Dq and hM4Di, respectively) are the most frequently used DREADDs. Owing to two-point mutations in the ligand binding domain, both receptors can be easily activated upon administration of synthetic compounds of which the most widely used is clozapine-N-oxide (CNO) (Armbruster et al., 2007; Wess et al., 2013). Pioneering studies from Fiacco and colleagues, using transgenic mice expressing the GPCR A1 (MrgA1) in astrocytes, showed that stimulation of MrgA1 elicited Ca^2+^ waves in astrocytes (Fiacco et al., 2007). However, this did not seem to influence neuronal excitability and synaptic plasticity. This lack of effect on neuronal function was met with disappointment by the field and MrgA1-based studies were generally discontinued in favor of those using DREADDs expressed in astrocytes, which were shown to modulate neuronal activity and hence impact animal physiology and behavior (Bang et al., 2016; Shen et al., 2021).

In GFAP-hM3Dq transgenic mice (Agulhon et al., 2013) and in mice injected in the visual cortex with AAV-GFAP-hM3Dq (Bonder and McCarthy, 2014), hM3Dq stimulation with CNO increases intracellular Ca^2+^ levels in astrocytes. The same response has also been described in hM3Dq-activated astrocytes in the hippocampus (Chai et al., 2017; Adamsky et al., 2018; Durkee et al., 2019), striatum (Chai et al., 2017), and nucleus accumbens core (Bull et al., 2014; Corkrum et al., 2020). In contrast, the exact signalling outcome of hM4Di-mediated astrocyte manipulation seems to be less evident as some studies have reported increased astrocytic Ca^2+^ concentrations (Chai et al., 2017; Durkee et al., 2019; Nagai et al., 2019), while others have shown a decrease (Yang et al., 2015; Xin et al., 2019), or even no difference (Nam et al., 2019). These distinct effects have been described between different brain regions (Chai et al., 2017) but also within the same region, like the hippocampus (Chai et al., 2017; Durkee et al., 2019; Kol et al., 2020). Astrocytes are known to be a molecularly and functionally heterogeneous cell population both between and within brain regions (Ben Haim and Rowitch, 2017; Khakh and Deneen, 2019; Pestana et al., 2020; Holt, 2023). Hence, these different responses could be potentially linked to the activation of molecularly distinct astrocyte subtypes. Indeed, work by Chai and colleagues revealed that hM3Dq expression and activation in both hippocampal and striatal astrocytes produces roughly equivalent increases in intracellular Ca^2+^, while the effects of hM4Di on Ca^2+^ were significantly higher in striatal astrocytes, again pointing to specific differences in intracellular signalling pathways (Chai et al., 2017).

Similar to the effects generated by the activation of endogenous GPCRs, several studies have shown that DREADD-induced Ca^2+^ increases result in gliotransmitter release from astrocytes, with subsequent effects on synaptic plasticity and function and, consequently, in behavior and processes such as learning and memory formation. hM3Dq-activated astrocytes in the rat nucleus accumbens core were reported to release glutamate, which was suggested to impact synaptic plasticity (Bull et al., 2014; Scofield et al., 2015). Other groups have reported the release of ATP from hM3Dq-activated astrocytes in the medial basal hypothalamus (Yang et al., 2015) and the nucleus accumbens core (Corkrum et al., 2020). Kang et al. showed that astrocytes in the dorsomedial striatum, activated using hM3Dq, release ATP which, once metabolized into adenosine, induces neuronal activity and, consequently, a shift from habitual to goal-directed seeking behaviors (Kang et al., 2020). Release of ATP from hM3Dq-activated astrocytes in the amygdala was shown to activate A_1A_ receptors, inhibiting neuronal activity and reducing fear behavior (Martin-Fernandez et al., 2017). Moreover, in astrocytes from the hippocampal CA1, hM3Dq-mediated activation induced astrocyte secretion of D-serine, which enhanced synaptic plasticity and memory formation (Adamsky et al., 2018). In an independent study, Durkee and colleagues found that stimulation of astrocytes using hM3Dq in the same brain region resulted in glutamate release, increasing neuronal excitability via NMDAR activation (Durkee et al., 2019). The effects of hM4Di activation on gliotransmitter release have been less explored. Evidence suggests that hippocampal astrocyte activation via hM4Di leads to glutamate release and a consequent increase in neuronal activity (Durkee et al., 2019; Nam et al., 2019). Overall, these studies highlight the role of astrocytes in complex animal behavior and function, while showcasing the incredible potential of this technique to further expand our knowledge of astrocyte-neuron interplay during processes such as memory formation.

Overall, these studies confirm that using Gi- and Gq-DREADDs to manipulate astrocytes seems to recapitulate, at least to a degree, their responses to endogenous neurotransmitters, resulting in neuronal modulation and behavioral effects. Thus, their use presents a potential entry point for uncovering new molecular pathways to manage CNS function. However, despite their incredible potential, it is important to acknowledge that using DREADDs also comes with its challenges and limitations. For example, it is unlikely that DREADD activation faithfully recapitulates all aspects of the highly complex Ca^2+^ signalling elicited under standard physiological conditions (Semyanov et al., 2020). In addition, when using viral vector-based delivery systems, the genomic titer of the vectors, multiplicity of infection in cells and relative promoter strength may limit DREADD expression levels. These factors could account, at least in part, for the distinct effects of DREADD activation observed in different studies. Furthermore, it has been reported that systemically administered CNO does not easily penetrate the blood-brain barrier and back-metabolizes into clozapine (Jann et al., 1994; Gomez et al., 2017). Since clozapine itself is a muscarinic agonist, it can activate endogenous receptors, potentially leading to off-target effects, as it has been observed in rats, mice and humans (Jann et al., 1994; MacLaren et al., 2016; Gomez et al., 2017; Bærentzen et al., 2019). Considering these issues, an effort has been made to develop new chemical compounds, such as Compound 21 (C21) and perlapine (Chen et al., 2015; Thompson et al., 2018), JHU37152 and JHU37160 (Bonaventura et al., 2019), and deschloroclozapine (Nagai et al., 2020; Nentwig et al., 2022). These molecules showcase high affinity and selectivity for DREADDs and, so far, minimal off-target effects have been identified. However, these novel compounds are still poorly characterized, and therefore, despite its obvious limitations, CNO remains the most commonly used compound for DREADD activation.

## Astrocytes as valid targets for CNS disease treatment

**FIGURE 2:**
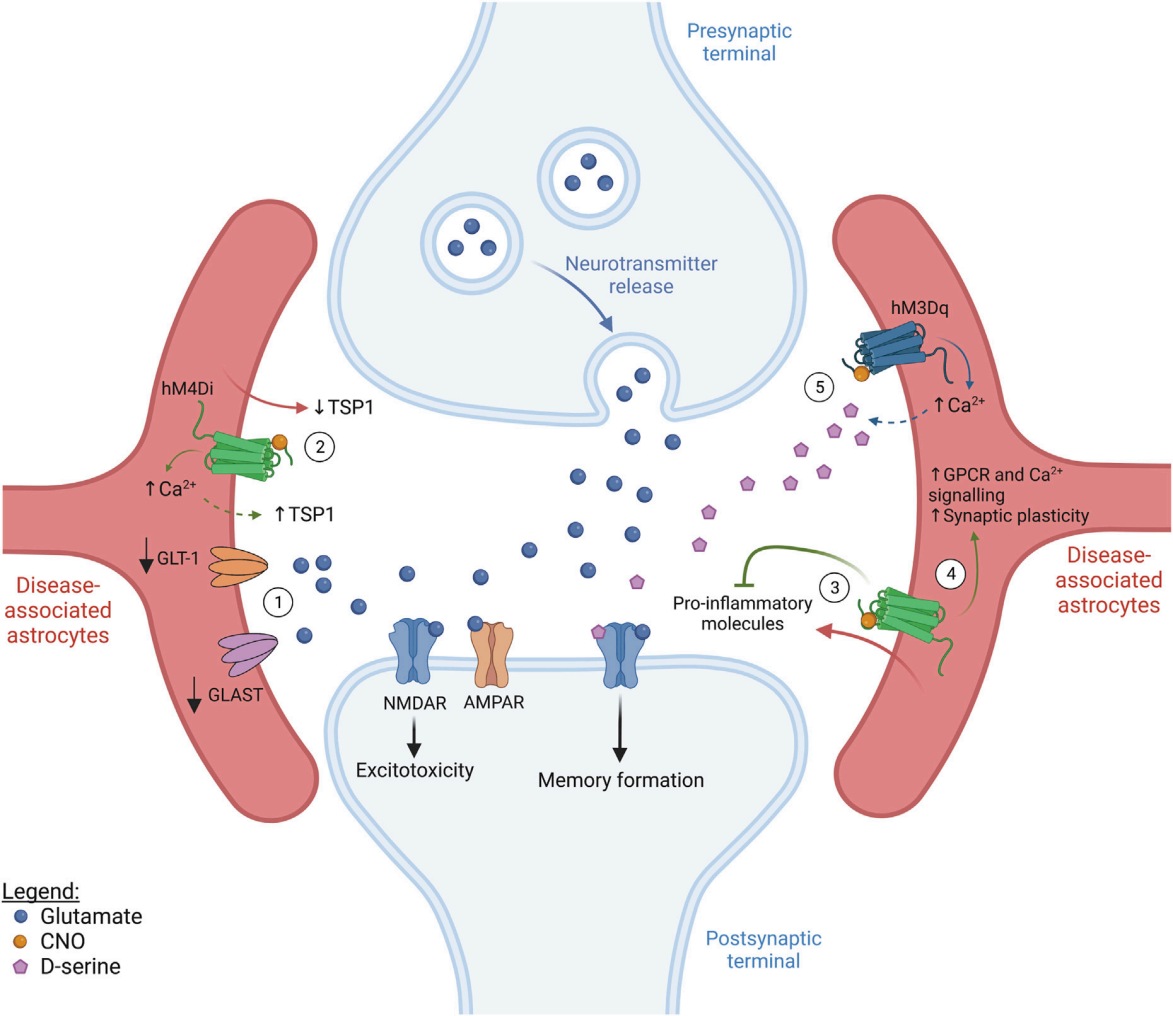
Targeting astrocyte activity in CNS disease (figure represents a general synapse). ① In several brain diseases, the surface expression of glutamate transporters, such as GLT-1 and GLAST, is significantly decreased. This compromises glutamate uptake from the synaptic cleft, leading to excitotoxicity and neuronal death. ② Most neurodevelopmental diseases show decreased spine density and, in some cases, astrocyte-derived thrombospondin (TSP1) secretion was shown to be decreased. Since astrocyte stimulation via hM4Di has been shown to induce elevations in intracellular Ca^2+^ as well as TSP1 release in the dorsal striatum, this represents a potential approach to promote structural synapse formation in patients with neurodevelopmental diseases, but beneficial behavioral outcome is still to be established. ③ Neuroinflammation is a common hallmark of neurodegenerative diseases and astrocytes are known to secrete pro-inflammatory molecules which contribute to inflammation propagation. Interestingly, hM4Di-mediated astrocyte activation in the CA1 was reported to suppress inflammation, resulting in an improvement in cognitive function. ④ Selective hM4Di stimulation in striatal astrocytes was also found to phenocopy GPCR activation by increasing Ca^2+^ signalling, rescuing astrocytic functional impairments and synaptic dysfunction associated with Huntington’s disease. ⑤ Selective activation of hM3Dq was shown to increase Ca^2+^ levels in astrocytes in the cingulate cortex and hippocampus. Increased Ca^2+^ in cortical astrocytes rescued neuronal activity and protected against seizures and day/night hyperactivity associated with early Alzheimer’s disease. Additionally, the increased Ca^2+^ levels driven by hM4Di activation in the hippocampus are thought to lead to D-serine release and improved memory formation. Solid arrows indicate established/tested effects, while dashed lines represent circumstantial/hypothetical links.

Neuronal circuit activity is easily disrupted as a consequence of synaptic dysfunction, a common hallmark of several neurological disorders, ranging from neurodevelopmental to neurodegenerative. Astrocyte dysfunction has also been reported in many CNS disorders over the years and is thought to contribute to disease mechanisms. Therefore, manipulating astrocytic activity and function at an early stage could hold potential for the development of novel approaches to decrease the severity and progression of the disease (Figure 2). It is interesting to note that categories of disorders present phenotypic commonalities. While the strategy to manage, for example, neurodevelopmental diseases could mainly focus on promoting astrocyte-mediated synaptogenesis, for other disorders one could prioritize re-establishing homeostatic astrocyte-neuron signalling and controlling chronic inflammation.

### Neurodevelopmental diseases

Neurodevelopmental disorders comprise a heterogeneous group of diseases deriving from impaired nervous system development. They are commonly associated with alterations in neuronal spine density and structure and synaptic function. Astrocyte dysfunction is often present and is commonly shown by decreased secretion of pro-synaptogenic factors and alterations in neurotransmitter clearance, affecting synaptic transmission. These phenotypes are observed in diseases such as Fragile-X and Down syndrome (Caldwell et al., 2022).

**Fragile-X syndrome (FXS)** patients possess an expansion in the trinucleotide CGG of the *FMR1* gene resulting in autistic traits, such as intellectual disability and social anxiety, abnormal behavior and high susceptibility to seizures (Penagarikano et al., 2007; Yudkin et al., 2014). The FMR protein (FMRP) is involved in spine maturation. Therefore, FXS patients typically display impaired synaptic development, with a high density of thin, immature spines and, consequently, defective synaptic activity (Rudelli et al., 1985; Hinton et al., 1991). In astrocyte-specific *Fmr1* knock-out mice, the expression of GLT-1 is decreased leading to impairment of glutamate reuptake and excitotoxicity (Figure 2) (Higashimori et al., 2016). *Fmr1* knockdown in cultured hippocampal neurons increases mGluR1/5-mediated signalling which drives the internalization of AMPARs, an important mechanism in mGluR-mediated LTD (Nakamoto et al., 2007). Enhanced mGluR-mediated LTD is observed in multiple brain regions of patients with FXS, such as the hippocampus, amygdala and cerebellum, leading to epileptic episodes (Chuang et al., 2005), increased anxiety (Suvrathan et al., 2010), and motor deficits (Koekkoek et al., 2005), respectively. Therefore, it would be of interest to direct future work towards unravelling whether potentiating astrocyte secretion of factors such as Gpc4 and Gpc6 could help with the recruitment of AMPARs back to synapses as a means of restoring neuronal activity.

**Down syndrome (DS),** also known as trisomy of chromosome 21, is the most common genetic form of mental disability (Antonarakis et al., 2020). Similar to FXS, in DS patients and mouse models of DS, spine density and structure are severely altered, compromising neuronal and synaptic plasticity, particularly in the cortex and hippocampus (Marin-Padilla, 1972; Benavides-Piccione et al., 2004). TSP1 levels are significantly decreased in cultured astrocytes from DS patients. In mixed cultures of DS-derived astrocytes and wild-type hippocampal neurons, this leads to perturbations in the development of dendritic spines, which were rescued by the addition of recombinant TSP1 to the culture medium (Garcia et al., 2010).

Given the common defects in spine and synaptic development observed in FXS and DS, it would be logical that targeting astrocyte function in order to promote the secretion of synaptogenic molecules in early development could be a potential way to alleviate disease phenotype. A study by Nagai and colleagues recently showed that, in the dorsal striatum, astrocyte activation using hM4Di *in vivo* stimulates the release of TSP1 by astrocytes (Figure 2) and increases excitatory synapse formation and the firing rate of medium spiny neurons (MSN) (Nagai et al., 2019). However, this increased TSP1 also led to behavioral abnormalities, including hyperactivity and disrupted attention. Despite this apparent contraindication, we would argue that it should be investigated whether TSP1 release is also stimulated by hM4Di-mediated astrocyte activation in other brain regions, such as the hippocampus, and what the exact functional and behavioral consequences of such manipulation would be. Exploring this is crucial to support or refute the idea of astrocytic TSP1 release as a therapeutical option for pathologies affecting different brain areas, since the effects of hM4Di manipulation could differ due to regional astrocyte heterogeneity. Additionally, research should further focus on determining whether DREADD-mediated astrocyte activation can also induce the release of other factors like Gpc4, Gpc6 and Chrdl1 to promote functional synapse maturation.

### Neurodegenerative diseases

**Alzheimer’s disease (AD)** is the most common neurodegenerative disease and patients clinically present cognitive decline and progressive dementia with loss of long-term memory. AD is primarily characterized by the accumulation of Aβ plaques and hyperphosphorylated tau (Long and Holtzman, 2019). Over the years, several studies have highlighted the importance of astrocyte (dys)function throughout the course of AD pathology. A comparison of single-nucleus RNA-seq data sets from the prefrontal cortex of patients with no- and early-AD pathology, aged around 87 years old, has shown that most transcriptomic changes already occur before the development of pathological hallmarks (Mathys et al., 2019). In the APPSwe/PS1dE9 mouse model, astrocytes display altered expression of genes associated with synaptic regulation from 4 months onward (Pan et al., 2020). In fact, a recent study by Shah and colleagues demonstrated decreased astrocytic Ca^2+^ signalling in the cingulate cortex of *App* ^*NL-G-F*^ mice aged 6–12 weeks, reflecting disrupted network activity in this brain area before any detectable Aβ plaque formation, just as in human AD patients (Shah et al., 2022). At the later stages of AD, reactive astrocytes emerge and secrete IL-1β, and TNF-α, among other pro-inflammatory cytokines (Osborn et al., 2016). Single-cell/nucleus RNA sequencing studies on entorhinal and prefrontal cortical astrocytes from AD patients, as well as on cortical astrocytes from 5XFAD and APPSwe/PS1dE9 mouse models, have revealed a high diversity of reactive astrocytes, not only associated with different brain regions but also with different stages of disease progression, highlighting the complexity of disease-associated astrocyte heterogeneity (Orre et al., 2014; Grubman et al., 2019; Mathys et al., 2019; Zhou et al., 2020). In addition, females were found to show higher transcriptional susceptibility to AD pathology, suggesting a gender-based astrocyte disease response (Mathys et al., 2019). Contrary to what was recently observed for the pre-symptomatic AD stage (Shah et al., 2022), Ca^2+^ signalling appears increased in astrocytes at late disease stages (6–9 months old) in the APPSwe/PS1dE9 mouse model (Kuchibhotla et al., 2009; Lines et al., 2022), and this has been linked to increased astrocyte reactivity (Shigetomi et al., 2019). Increased Ca^2+^ signalling typically enhances the release of gliotransmitters, such as glutamate (Perea et al., 2009). Additionally, GLT-1 expression is decreased in the cortex (Scott et al., 2011), inferior parietal lobe (Lauderback et al., 2001), and hippocampus and gyrus frontalis medialis (Jacob et al., 2007) of human patients. This decrease in GLT-1 was also observed in the cortex and hippocampus of 8- and 18-month-old AβPP23 mice (Schallier et al., 2011), and is thought to underlie the neuronal hyperactivity seen in this mouse model and AD in general. hM3Dq-mediated astrocyte activation rescued astrocytic Ca^2+^ signalling in the cingulate cortex of pre-symptomatic *App* ^*NL-G-F*^ mice and, consequently, neuronal activity and functional connectivity of brain circuits. This also prevented typical symptoms presenting at the early stages of AD, like seizures and day/night hyperactivity, emphasizing that astrocytes are likely major players in early AD (Figure 2) (Shah et al., 2022). On the other hand, when, in the same study, astrocytes from control animals were similarly activated through hM3Dq, the increase in Ca^2+^ signalling was much more pronounced and induced neuronal hyperactivity (Shah et al., 2022). DREADD activation of healthy and disease-associated astrocytes thus causes opposing effects on neuronal activity, producing distinct functional and behavioral outcomes. This implies that the use of astrocyte activation therapeutically in the clinic will have to be done cautiously and will likely need matching to reactivity status (see below).

From work in normal mice, DREADD-mediated astrocyte manipulation leading to ATP and D-serine release, of potential relevance to the AD field, has been reported. Astrocyte activation in the hippocampus, using hM4Di, decreases Ca^2+^ signalling and astrocyte-secreted ATP, compromising long-term, but not short-term, memory (Kol et al., 2020). hM3Dq-mediated stimulation of hippocampal astrocytes induces Ca^2+^ waves, leading to D-serine release from astrocytes, which facilitates memory formation (Adamsky et al., 2018) (Figure 2). Given the positive impact of these DREADD manipulations on memory formation and retention, we further propose investigating the functional and behavioral outcomes of similar astrocyte-specific, DREADD-mediated manipulations in the specific context of AD. Furthermore, understanding the dynamics of how astrocytic phenotypes adapt throughout pathology progression and testing how these astrocyte populations respond to DREADD activation will be crucial in allowing their specific targeting to obtain the best possible therapeutic outcome. Additionally, this will likely require the simultaneous development of novel biomarker assays that allow accurate assessment of the temporal progression of the disease.

**Huntington’s disease (HD)** is caused by an extension of the CAG repeat in the huntingtin gene, leading to a wide variety of motor, psychiatric and cognitive symptoms. Mutant huntingtin forms aggregates and leads to astrocyte dysfunction and neurodegeneration, particularly in the cortex and the striatum (Bates et al., 2015; Ghosh and Tabrizi, 2018). Gene expression studies on human and mouse HD samples have focused on unravelling the diversity of reactive astrocytes in HD (Liddelow et al., 2017; Diaz-Castro et al., 2019; Al-Dalahmah et al., 2020). For instance, a study conducted on striatal astrocytes from pre-clinical HD mouse models and patients found that these astrocytes commonly show reduced expression of genes mostly related to GPCR, Ca^2+^ and glutamate signalling (Diaz-Castro et al., 2019). The importance of GPCR signalling in HD pathology has been recently highlighted by the demonstration that *in vivo* stimulation of striatal astrocytes using hM4Di was able to rescue astrocytic function, including astrocyte-mediated synaptogenesis, and synaptic and behavioral phenotypes characteristic of HD pathology (Yu et al., 2020b). hM3Dq-mediated astrocyte stimulation in the striatum of normal mice evoked increases in astrocytic Ca^2+^ (Chai et al., 2017). Thus, this could also be a valid approach to further explore the effects of modulating Ca^2+^ signalling in HD astrocytes, just as in AD, to reveal novel ways of managing such diseases (Figure 2).

**Multiple sclerosis (MS)** is a chronic inflammatory disease characterized by axonal demyelination leading to motor deficits (Filippi et al., 2018). In MS, astrocytes show complex dynamics, which are central to the disease’s progression. Although the elimination of reactive astrocytes at early stages in a mouse model of MS worsens neuroinflammation and disease severity (Liedtke et al., 1998; Toft-Hansen et al., 2011), if such depletion is restricted to advanced, chronic phases of MS, disease pathology is improved (Mayo et al., 2014). Analysis of astrocyte diversity in MS has identified a neurotoxic astrocyte subtype characterized by complement component 3 expression (Liddelow et al., 2017), as well as an anti-inflammatory astrocyte subtype, which seems to limit pathology (Sanmarco et al., 2021). Together, this suggests that some astrocytes, likely with an anti-inflammatory phenotype, might be important in the initial stages of the disease but, in the long term, neurotoxic subtypes might take over, aggravating disease pathology. Therefore, it appears that neuroinflammation should be handled in a temporal- and/or astrocyte subtype-specific manner. A recent study by Kim et al. has revealed that hM4Di-mediated astrocyte stimulation, in the hippocampal CA1 region, is capable of suppressing LPS-induced neuroinflammation, suggesting that hM4Di-activation engages anti-inflammatory mechanisms (Kim et al., 2021) (Figure 2). Exploring how DREADDs modulate neuroinflammation might identify anti-inflammatory pathways which can be exploited therapeutically to manage MS, and perhaps other neurodegenerative diseases characterized by a large inflammatory component. Using DREADDs to achieve this is a particularly attractive option, since receptor expression could potentially be restricted to neurotoxic astrocytes, through the use of astrocyte subtype-specific promoters. Even though these promoters are not available yet, characterization of astrocyte heterogeneity in MS might pave the way to implement such a targeted strategy, thus revealing how DREADDs differentially affect different subtypes of reactive astrocytes. As DREADD activation can also be temporally controlled this further provides the opportunity of exploring DREADD-mediated effects at specific disease stages, which will be particularly important in chronic (relapsing) conditions.

### Other neurological conditions

Besides intrinsic factors, such as genetic mutations, imbalanced CNS function can be induced by external factors, like drug abuse, which also disrupt homeostasis and compromise synaptic transmission. It is important to note that even in scenarios like addiction, in which astrocytic function does not appear fully compromised, modulating astrocyte activity via DREADD stimulation can present beneficial effects for neuronal function and circuit homeostasis.

**Addiction** relates to a loss of control driving consumption of certain substances, such as alcohol or drugs. It appears such behavior is elicited by imbalances in glutamatergic signalling, mainly in the prefrontal cortex and striatum (Kalivas, 2009; Stefanik et al., 2013). For example, in animal models, basal levels of extracellular glutamate are decreased in the nucleus accumbens following cocaine exposure, reducing mGluR2/3 stimulation (Baker et al., 2003; Moussawi and Kalivas, 2010; Reissner and Kalivas, 2010). However, during drug-seeking or reinstatement behavior, rats display increased synaptic activity in the connections between the prefrontal cortex and nucleus accumbens, leading to high levels of glutamate release (Scofield and Kalivas, 2014). This is compounded by the fact that prior exposure of rats to cocaine negatively impacts levels of GLT-1 expression in nucleus accumbens astrocytes, thus reducing extracellular glutamate uptake, and leading to excessive accumulation of glutamate at the synapse. When combined with a reduced basal level of signalling, this acts to ‘hyperactivate’ the system, and it is this enhanced level of glutamatergic signalling which has been proposed to increase susceptibility to continuous relapses (Kalivas, 2009). Crucially, in this respect, a study by Scofield and colleagues, conducted in mice, demonstrated that hM3Dq astrocyte activation in the nucleus accumbens, before the start of a reinstatement period, elicits astrocytic glutamate release. This re-establishes glutamate homeostasis previously disrupted by exposure to cocaine and restores mGluR2/3 tone, acting to inhibit cocaine-seeking relapse (Scofield et al., 2015) and highlighting the possibility of using astrocyte-based strategies to treat addiction.

**Sensory loss** can develop because of an injury or age-related pathology to sensory organs, such as the retina in the eyes. Partial or complete loss of a particular sense deprives the brain region(s) that were involved in processing the lost sense of their inputs, thus compromising neuronal activity. However, it is now recognized that these brain areas do not become silent zones. Instead, they are reactivated and become responsive to stimuli from the spared senses (Pascual-Leone et al., 2005; Hahamy and Makin, 2019). Evidence of this is observed, for example, in blind patients. Following vision loss, visual cortical areas become actively involved in discriminating somatosensory stimuli during Braille reading (Sadato et al., 1996; Leo et al., 2012). Considering the close association between astrocytes and synapses, it is not surprising that they appear involved in driving the required neuroplasticity through various mechanisms. We have recently demonstrated that hM4Di-mediated astrocyte activation in the visual cortex is capable of boosting such neuronal reactivation following sudden partial vision loss in adult mice (Hennes et al., 2020). The exact mechanisms underlying these effects, however, remain unclear. Exploring the downstream effects of hM4Di-mediated astrocyte activation in this monocular deprivation model might bring new insights into mechanisms of neuroplasticity, supporting the idea that DREADD-based astrocyte manipulation may have great potential for therapy development to treat patients with late-onset sensory loss.

## Concluding remarks

Altogether, these studies highlight the central role of astrocyte dysfunction, and consequent synaptic dysfunction, in several CNS pathologies and thus the essential contribution of astrocytes to normal circuit development and function. Given the importance of GPCR signalling for astrocyte activity, we propose that expressing genetically engineered GPCRs in astrocytes could be a promising strategy to identify relevant signalling pathways that could ameliorate brain dysfunction occurring after injury or throughout disease. The potential application of DREADDs across the spectrum of conditions lies in the range of downstream effects triggered upon astrocyte stimulation, which most notably includes the release of distinct neuroactive molecules that differentially modulate synaptogenesis and neuronal activity. The positive impact of astrocyte manipulation using DREADDs on synaptic transmission, cognitive function and behavior, mainly by eliciting astrocytic Ca^2+^ waves and increasing the release of TSP1, ATP, D-serine or glutamate, has been demonstrated by several groups. Even though this holds potential to manage brain disease, direct evidence of DREADD-mediated astrocyte activation leading to the improvement of disease pathology is still scarce.

Most CNS pathologies share common features like loss of spine density, impaired glutamate clearance and synaptic transmission, and neuroinflammation. Still, the causative factor(s) for these diseases differ widely. Genetic mutations, the cellular and molecular environment characteristic of each disease, as well as the brain regions and specific neuronal circuits affected, differ between diseases. Therefore, manipulating astrocyte activity in these different contexts may very well lead to different functional consequences, which could either improve or worsen the disease state. Thus, future research should focus on assessing the effects of astrocyte activity modulation in each specific disease, and probably also needs to acknowledge the issue of disease time-course (see below). Furthermore, astrocyte heterogeneity is likely a contributing factor in determining the specific outcome of DREADD manipulation. The complexity of this topic increases even more when considering that new astrocyte subtypes characteristic for each particular disease, and even disease stage-specific astrocytes, usually arise. Identifying and differentiating the molecular profile of such disease-associated astrocytes may allow the design of astrocyte subtype-specific promoters to target DREADD vectors to maleficent astrocyte subpopulations. We also propose that conducting sequencing studies on DREADD-activated astrocytes could be highly relevant to fully understand the effects of this type of stimulation on the molecular fingerprint of the cells. This could bring insights into whether astrocytes respond by boosting endogenous signalling pathways, or by triggering new ones, generating insights into potential signalling pathways that can be exploited therapeutically. Another key aspect to take into consideration is that mouse and human astrocytes are morphologically, transcriptionally, and functionally different (Oberheim et al., 2006; Li et al., 2021). Using, for example, human cerebral organoids (Dezonne et al., 2017) would be a valuable strategy to test how human astrocytes are affected by DREADDs and help in the transition from the bench to the clinic.

It is interesting to note that very few studies so far have demonstrated inhibition of astrocytic Ca^2+^ waves following DREADD activation. Most studies also report an increase in TSP1 or gliotransmitter release, particularly glutamate, upon DREADD-mediated astrocyte stimulation. However, some pathologies display increased TSP1 release (Krencik et al., 2015), enhanced Ca^2+^ signalling in astrocytes or glutamate accumulation at the synapse, thus suggesting that use of the existent DREADD receptors might not be a good strategy to unravel possible ways to manage such diseases, due to undesirable, or even toxic, effects. For instance, excessive TSP1 release might induce the overproduction of new synapses, which may further disrupt neuronal circuits, while excessive glutamate release can lead to excitotoxicity. It is also important to point out that Ca^2+^ is likely not the only second messenger influenced by DREADDs. To what extent DREADDs are also acting on alternative, Ca^2+^-independent signalling pathways affecting, for example, cAMP levels remains unclear. Exploring the impact of DREADD-evoked astrocyte activation on other signalling pathways, all of which may impact synapse formation and function, most likely depends on the development of new and more sensitive tools. This will hopefully create a comprehensive understanding of the biological effects of DREADD activation on cells, leading to a deeper mechanistic understanding of cell function and insights into disease, which will ultimately allow the development of ‘next-generation’ therapeutics.
